# Advanced-age C57BL/6JRj mice do not develop obesity upon western-type diet exposure

**DOI:** 10.1080/21623945.2019.1590893

**Published:** 2019-03-26

**Authors:** Ellen Vercalsteren, Christine Vranckx, Liesbeth Frederix, Marleen Lox, H. Roger Lijnen, Ilse Scroyen, Bianca Hemmeryckx

**Affiliations:** Center for Molecular and Vascular Biology, Department of Cardiovascular Sciences, KU Leuven, Leuven, Belgium

**Keywords:** Aging, C57BL/6J mice, western-type diet, obesity

## Abstract

Obesity has become a global health-threat for every age group. It is well known that young mice (10-12 weeks of age) fed a western-type diet (WD) become obese and develop higher cholesterol levels and liver steatosis whereas insulin sensitivity is reduced. Less is known, however, about the effect of a WD on advanced-age mice. Therefore, 10 week-old (young) and 22 month-old (advanced-age), male C57BL/6JRj mice were kept on either a WD or a control diet (SFD) for 15 weeks. In contrast to young mice, advanced-age mice on WD did not show a higher body weight or adipose tissue (AT)-masses, suggesting a protection against diet-induced obesity. Furthermore, plasma adiponectin and leptin levels were not affected upon WD-feeding. A WD, however, did induce more hepatic lipid accumulation as well as increased hepatic expression of the macrophage marker F4/80, in advanced-age mice. There were no significant differences in mRNA levels of uncoupling protein-1 or F4/80 in brown AT (BAT) or of several intestinal integrity markers in colon suggesting that the protection against obesity is not due to excessive BAT or to impaired intestinal absorption of fat. Thus, advanced-age mice, in contrast to their younger counterparts, appeared to be protected against diet-induced obesity.

## Introduction

Obesity has become a severe and worldwide health problem, not only for the young and adult population but also for the elderly. This is important since the world population is ageing rapidly. Therefore, interest in healthy ageing is increasing exponentially. Obesity is defined as an excessive or abnormal fat accumulation as a result of a daily positive energy balance. To study diet-induced obesity, C57BL/6J mice are frequently used [1]. Young C57BL/6J mice (4 weeks of age) become obese and develop hyperglycaemia and hyperinsulinaemia after 16 weeks exposure to a western-type diet (WD, high in fat and carbohydrate content) [2]. Furthermore, adult C57BL/6J mice (8 weeks old) fed a high-fat diet (HFD) become obese, associated with higher fasting glucose and insulin levels and a reduction in glucose tolerance and insulin sensitivity [3]. In addition, total cholesterol levels are elevated and enhanced liver steatosis develops [4]. Previous work published by our lab demonstrated that even without WD-feeding, 12 month-old mice had a higher body weight as well as increased subcutaneous (SC) and gonadal (GN) adipose tissue (AT) weights compared to their 10 week-old counterparts [5]. Less is known, however, about the effect of a WD on advanced-age mice (between 22 and 26 months). As the average lifespan of C57BL/6JRj mice is approximately two years, 22 month-old mice are adequate to study the effect of a WD on elderly animals. Thus, the aim of this study is to investigate the effect of a WD on the development of obesity, insulin resistance and hepatic steatosis in advanced-age mice.

## Materials and methods

### Animal model

Male C57BL/6JRj mice at the age of 6 weeks (n = 10) and 21 months (n = 20) were purchased from Janvier. All animals were co-housed (2–3 animals/cage) in micro-isolation cages in a temperature-controlled environment (22°C) on a 12-h night/day cycle with *ad libitum* access to drinking water and standard chow (Sniff® R/M-H, Sniff Spezialdiäten). Animals were allowed to acclimatize in our facility for 4 weeks. After fasting for 6 h in the morning glucose levels were determined in blood retrieved on acid citrate from the retro-orbital sinus of 2%-isoflurane-anaesthetized mice. Mice were divided into four groups (n = 5 per group for 10-week old mice (= young groups) and n = 10 per group for 22 month-old mice (= advanced-age groups)) with a similar baseline body weight and blood glucose level. Subsequently, one group of each age received a WD (high in sugar and fat (43% kJ carbs, 42% kJ fat and 15% kJ protein); 19.1 kJ/g; TD88137; Ssniff) for 15 weeks, while the other two groups were exposed for 15 weeks to a control standard-fat diet lower in carb- and fat content (SFD; 30% kJ carbs, 30% kJ fat and 40% kJ protein; 17.2 kJ/g; Ssniff) (Table 1). Body weight and food intake were monitored weekly. After 15 weeks, mice were fasted for 6 h in the morning and blood glucose levels were measured. Mice were euthanized by intraperitoneal (ip) injection of 60 mg/kg sodium pentobarbital (Dolethal), and blood was obtained as described above. The anaesthetized mice were first injected with heparin (ip 16 IU/g body weight) to prevent clotting and subsequently perfused with saline to remove blood from the organs. Subcutaneous (SC), gonadal (GN), mesenteric (MES), brown adipose tissue (BAT), colon and faeces were isolated and weighed. SC, GN and MES fat, liver and colon samples were used for histology, RNA and protein analysis. All animal procedures were approved by the Ethical Committee of the KU Leuven (P031/2017) and performed in accordance with the NIH Guide for the Care and Use of Laboratory Animals (1996).

**Table 1.** Composition of the diets.

	SFD	WD
% Protein	40	15
% Fat	30	43
% Carbohydrates	30	42
**Detailed composition**		
casein (%)	46.850	19.500
sucrose (%)	19.624	33.404
Corn starch, pre-gelatinized (%)	9.000	15.000
Cellulose powder (%)	5.000	5.000
DL-Methionine (%)	0.300	0.300
Vitamins (%)	1.100	1.100
Minerals and trace elements (%)	4.300	4.300
Choline CI (50%)	0.200	0.200
Butylated hydroxytoluene (%)	0.010	0.010
Cholesterol (≥96%)	0.156	0.156
Butter fat (%)	12.460	20.000
Safflower oil (%)	0.800	0.800
linseed oil (%)	0.200	0.200

Detailed composition of the diets used for experiments. SFD = standard-fat diet, WD = western-type diet.

### Analyses

Blood glucose concentrations were measured using Glucocard strips (28,350, Menarini Diagnostics) and plasma triglyceride and cholesterol were evaluated using routine clinical assays. Plasma insulin (Mercodia), adiponectin (R&D Systems) and leptin (DY498, R&D Systems) levels were measured using commercially available ELISAs. The Homeostatic Model Assessment of Insulin Resistance (HOMA-IR-) index was calculated as follows: fasting glucose levels (mM) x fasting insulin levels (mU/L)/22.5. The content of triglycerides in the liver and BAT was quantified with the triglycerides FS* kit (Diasys), after preparation of liver tissue as described previously [6]. Briefly, 30 mg tissue was incubated overnight at 55°C in alcoholic KOH (2:3 60 ml EtOH 99% + 30 ml 30% KOH) until digestion was complete. The next day, 1 M MgCl_2_ (1:1, vol/vol) was added to the digested samples. After 10 min of incubation on ice and subsequent centrifugation, the supernatant was aspirated and analysed using the triglycerides FS* kit (Diasys). Lactate dehydrogenase (LDH) levels were assessed using the LDH assay kit (Abcam) according to the manufacturer’s instructions.

### Western blot

A Western blot for uncoupling protein-1 (UCP-1) was performed as described previously [7]. Briefly, BAT adipose tissue was homogenized using the FastPrep ribolyser (MP Biomedicals) in a buffer consisting of 10 mM Na phosphate pH 7.2, 150 mM NaCl, 1% Triton, 0.1% sodium dodecyl sulfate (SDS), 0.5% Na deoxycholate, 0.2% NaN_3_ and a protease inhibitor cocktail (Thermo Fischer Scientific). The BCA protein assay (Pierce) was used to determine protein concentration. An equal amount of protein was then loaded onto a 10% SDS-PAGE. Gels were transferred onto a 0.45 µm nitrocellulose membrane and blocked in 5% non-fat dry milk (Bio-Rad) in 10 mM Tris-HCl buffer with 150 mM NaCl and 0.5% Tween 20 pH 7.6 for 3 h. Hereafter, the membrane was probed with a UCP-1 antibody (Sigma-Aldrich). For the loading control, β-actin was used. A horseradish peroxidase-conjugated secondary antibody in TBST containing 5% non-fat dry milk was then added. Signals were detected using enhanced chemiluminescence (Thermo Fischer Scientific). Analysis was performed with Image J (NIH).

### Real-time PCR

TaqMan gene expression assays (Thermo Fischer Scientific Inc.) were used to analyse mRNA levels of several genes in liver and colon tissue and BAT by quantitative RT-PCR (Table 2), according to a protocol described previously [8]. Briefly, RNA extraction from tissues was performed using the RNeasy mini kit (Qiagen, Basel, Switzerland) according to the manufacturer’s instructions. Ten nanograms/microlitre RNA was reverse transcribed into cDNA using the Multiscribe^TM^ Reverse Transcriptase kit (Life Technologies, Thermo Fischer Scientific Inc.) according to the manufacturer’s protocol. The ABI 7500 Fast Sequence Detector was used to perform quantitative RT-PCR. Data were obtained as cycle threshold (Ct) values and normalized to the housekeeping gene β-actin (∆Ct = Ct_target_ – Ct_β-actin_). For each gene, two samples were analysed and the average Ct-value was calculated and used for analysis. Fold differences in gene expression were calculated with the ΔΔCt method, using C57BL/6J mice kept on SFD as calibrator.

**Table 2.** Markers detected via qPCR using TaqMan gene expression assays.

Gene	Assay
*CD36*	Mm_00432403_m1
*Timp-1*	Mm_00441818_m1
*Col1α1*	Mm_00801666_m1
*F4/80*	Mm_00802529_m1
*Fas*	Mm_00433237_m1
*Occln*	Mm_00500912_m1
*Zo-1*	Mm_00493699_m1
*Zon*	Mm_00516884_m1
*Ucp-1*	Mm_01244861_m1
*β-Actin*	Mm_01205647_g1

All assays were purchased from Life Technologies (Thermo Fischer Scientific Inc.). qPCR = qualitative polymerase chain reaction. CD = cluster of differentiation, Timp-1 Tissue Inhibitor of Matrix Metalloproteinase-1, Col1α1 = collagen type 1 α 1, Fas = TNF receptor superfamily member 6, Occln = occludin, Zo-1 = zona occludens-1, Zon = zonulin, Ucp-1 = uncoupling protein-1.

### Histology and microscopy

SC, GN and MES fat and liver samples were fixed for 72 h in 4% formaldehyde, washed with phosphate-buffered saline and paraffin embedded. Subsequently, 7 µm sections for fat and 4 μm sections for liver tissues were deparaffinized and stained with haematoxylin/eosin (H&E). Images of H&E-stained fat and liver sections were obtained using an inverted Axiovert 200M Zeiss microscope with normal light and the Axiovision Rel. 4.8.2 (Zeiss) at x100 magnification. Size and density of adipocytes were then determined on H&E-stained fat sections as described previously [9]. Briefly, 10 areas were calculated for each mouse using computerized image analyser. Subsequently, spherical morphometry was assumed to calculate the cell surface. Liver steatosis was assessed on H&E-stained hepatic sections as reported previously [10].

### Statistical analyses

Data are shown as means ± SEM for the number of animals studied. Differences between groups were analysed using the Mann-Whitney-U test. Values of p < 0.05 were considered statistically significant. The statistical analyses were performed with GraphPad Prism 7 software (GraphPad).

## Results

### Advanced age mice do not develop obesity upon western diet feeding

After 15 weeks of diet, one animal from the SFD-advanced-age group was excluded from the study, as it presented several pathologies including hepatomegaly, destruction of the left kidney and severe infection of the seminal vesicles glands. All other animals of the advanced-age group only suffered from minor age-related comorbidities such as infected seminal glands. In accordance with existing literature [11–14], we found that after 15 weeks of diet-exposure young mice fed a WD weighed significantly more compared to the control mice fed a SFD (p < 0.001) (Table 3). Body weight gain also was significantly higher in the young WD-fed group as compared to the SFD-fed young group. Between the advanced-age groups, however, no significant difference in body weight was detected (p = 0.196) (Table 3). We also analysed age-dependent effects and found that the SFD-fed young mice had gained significantly more weight compared to the advanced-age SFD-group (p = 0.032). In addition, the young mice on WD gained more weight as compared to their advanced-age counterparts (p = 0.001). No statistical difference in caloric intake was found between groups (p = 0.114 for the advanced-age and p = 0.486 for the young group, respectively) (Table 3).

**Table 3.** Body weight and organ weights.

	Young		Advanced-age	
	SFD	WD	SFD	WD
*n*	5	5	9	10
Body weight (g)				
Start	26 ± 0.82	26 ± 0.54	31 ± 0.69	31 ± 0.80
End	39 ± 1.1	49 ± 0.86 ***	41 ± 1.1	43 ± 1.8
Body weight gain (g)	13 ± 0.7	23 ± 0.67 **	9.8 ± 0.93 °	12 ± 1.6 °°
Caloric intake (kcal/day)	16 ± 0.3	16 ± 0.1	15.5 ± 0.48	19.9 ± 2.06
Organ weights (mg)				
SC adipose tissue	780 ± 46	1591 ± 54 **	789 ± 139	858 ± 140 °°
GN adipose tissue	2011 ± 203	2941 ± 114 **	1552 ± 207	1974 ± 270 °
MES adipose tissue	764 ± 93	1277 ± 29 **	612 ± 114	691 ± 101 °
Liver	1915 ± 202	3848 ± 342 **	1707 ± 84	2287 ± 249 * °
Brown adipose tissue	138 ± 11	278 ± 8 *	103 ± 10.7 °	152 ± 13.3 * °°°
Adipocyte size (μm^2^)				
SC	2753 ± 222	3550 ± 201	2720 ± 501	2425 ± 338
GN	3324 ± 422	4727 ± 340 *	2411 ± 287	2108 ± 121 °°°
MES	2624 ± 169	3366 ± 208	1584 ± 250	1684 ± 152 °
Adipocyte density (x10^−6^/μm^2^)				
SC	391 ± 39	287 ± 17 *	493 ± 94	525 ± 82 °
GN	327 ± 52	220 ± 16 *	475 ± 52	499 ± 32 °°°
MES	404 ± 30	307 ± 21	675 ± 90	654 ± 81 °

Body weight and organ weights of young and advanced-age mice kept on either a SFD or a WD for 15 weeks. * = *​*p < 0.05, ** = p < 0.01 and *** = p < 0.001 in SFD vs WD in either young or advanced-age mice. ° = p < 0.05, °° = p < 0.01 and °°° = p < 0.001 between young and advanced-age mice kept on the same diet. SFD = standard-fat diet, WD = western-type diet, SC = subcutaneous, GN = gonadal, MES = mesenteric.

SC, GN and MES AT-depots of young mice fed a WD were significantly larger compared to those of mice fed a SFD (p = 0.008 for all depots). However, in the advanced-age group, no statistical differences were found in AT-masses (p = 0.736 for SC, p = 0.243 for GN and p = 0.515 for MES). In addition, we found that all AT-depots of young WD-fed mice weighed significantly more as compared to those of the advanced-age WD-group (p = 0.008 for SC, p = 0.040 for GN and p = 0.013 for MES). Furthermore, adipocytes were significantly larger in GN AT of young WD-fed mice compared to SFD-fed mice (p = 0.032) but not in SC and MES AT (p = 0.056 and p = 0.064, respectively) (Table 3). Adipocyte density was significantly lower in young mice on WD compared to mice on SFD in SC and GN AT but not in MES AT (p = 0.032, p = 0.046 and p = 0.064, respectively). In contrast, in the advanced-age group, no statistical differences were found between diets in adipocyte size and density (Table 3). Interestingly, we did find that adipocyte size in the GN and MES AT of WD-fed advanced-age mice was significantly smaller than that of young WD-fed mice (p = 0.016 and p < 0.001, respectively). Furthermore, we found that in all three AT-depots, adipocyte density was higher in the WD-advanced-age group as compared to the WD-young group (p = 0.04 for SC, p < 0.001 for GN and p = 0.016 for MES). Taken together, these results suggest that, in contrast to young mice, advanced-age mice exposed to a WD do not develop obesity.

### A western-type diet affects liver function and metabolism in both young and advanced-age C57BL/6J mice

In both age groups, blood glucose and plasma insulin levels were not significantly different, resulting in a comparable HOMA-index (p = 0.095 for young and p = 0.386 for advanced-age mice, respectively) (Table 4). In the young WD-fed mice, plasma insulin, as well as the resulting HOMA-index, was significantly higher compared to their advanced-age counterparts (p = 0.019 for insulin and p = 0.001 for HOMA-index) (Table 4). Plasma cholesterol levels were significantly higher in mice on WD in both age groups (p = 0.008 for the young and p = 0.041 for the advanced-age mice) (Table 4). Additionally, we found that in both advanced-age groups the plasma cholesterol levels were significantly lower as compared to the young groups (p = 0.044 for the SFD and p = 0.002 for the WD). In the young mice, plasma leptin levels were significantly increased in the WD group compared to the SFD group (p = 0.008), whereas no differences were found in plasma adiponectin levels (p > 0.999) (Table 4). In the advanced-age group plasma leptin and adiponectin levels were similar in both groups (p = 0.211 and p > 0.999). Furthermore, plasma leptin was significantly higher in the young WD-fed group as compared to the advanced-age WD-fed group (p = 0.001), again indicating that a WD does not induce obese AT in advanced-age mice (Table 4).

**Table 4.** Metabolic parameters.

	Young		Advanced-age	
	SFD	WD	SFD	WD
*n*	5	5	9	10
Blood glucose (mg/dL)	165 ± 11	168 ± 4.9	129 ± 5.6	147 ± 8.6
Insulin (ng/mL)	2.2 ± 0.7	3.4 ± 0.3	1.5 ± 0.45	2.1 ± 0.55 °
HOMa-IR-index (mM x mU/L)	27 ± 8.5	48 ± 3.1	15 ± 4.4	21 ± 4.7 °
Total cholesterol (mg/dL)	101 ± 8.7	192 ± 17 **	66 ± 6.1 °	105 ± 11 * °°
Leptin level (ng/mL)	9.0 ± 2.4	30 ± 1.1 **	4.4 ± 1.8	8.8 ± 2.3 °°°
Adiponectin level (μg/mL)	6.1 ± 2.4	6.4 ± 4.0	6.8 ± 0.50	6.1 ± 0.45
Triglycerides (mg/dL)	46 ± 2.6	37 ± 2.4 *	33 ± 2.4 °°	27 ± 0 * °°
Alkaline phosphatases (U/L)	39 ± 5.8	112 ± 22 *	39 ± 2.0	48 ± 4.3 °°
ALT (U/L)	52 ± 3.2	188 ± 46 *	48 ± 3.7	135 ± 33
AST (U/L)	88 ± 3.2	146 ± 28	85 ± 5.4	160 ± 38

Metabolic parameters in plasma of young and advanced-age mice kept on either an SFD or a WD for 15 weeks. All metabolic parameters, except for glucose, were analysed in plasma samples. * = p < 0.05 and ** = p < 0.01 in SFD vs WD in young or advanced-age mice. ° = p < 0.05, °° = p < 0.01 and °°° = p < 0.001 between young and advanced-age mice kept on the same diet. AST = aspartate aminotransferase and ALT = alanine aminotransferase.

**Figure 1.**
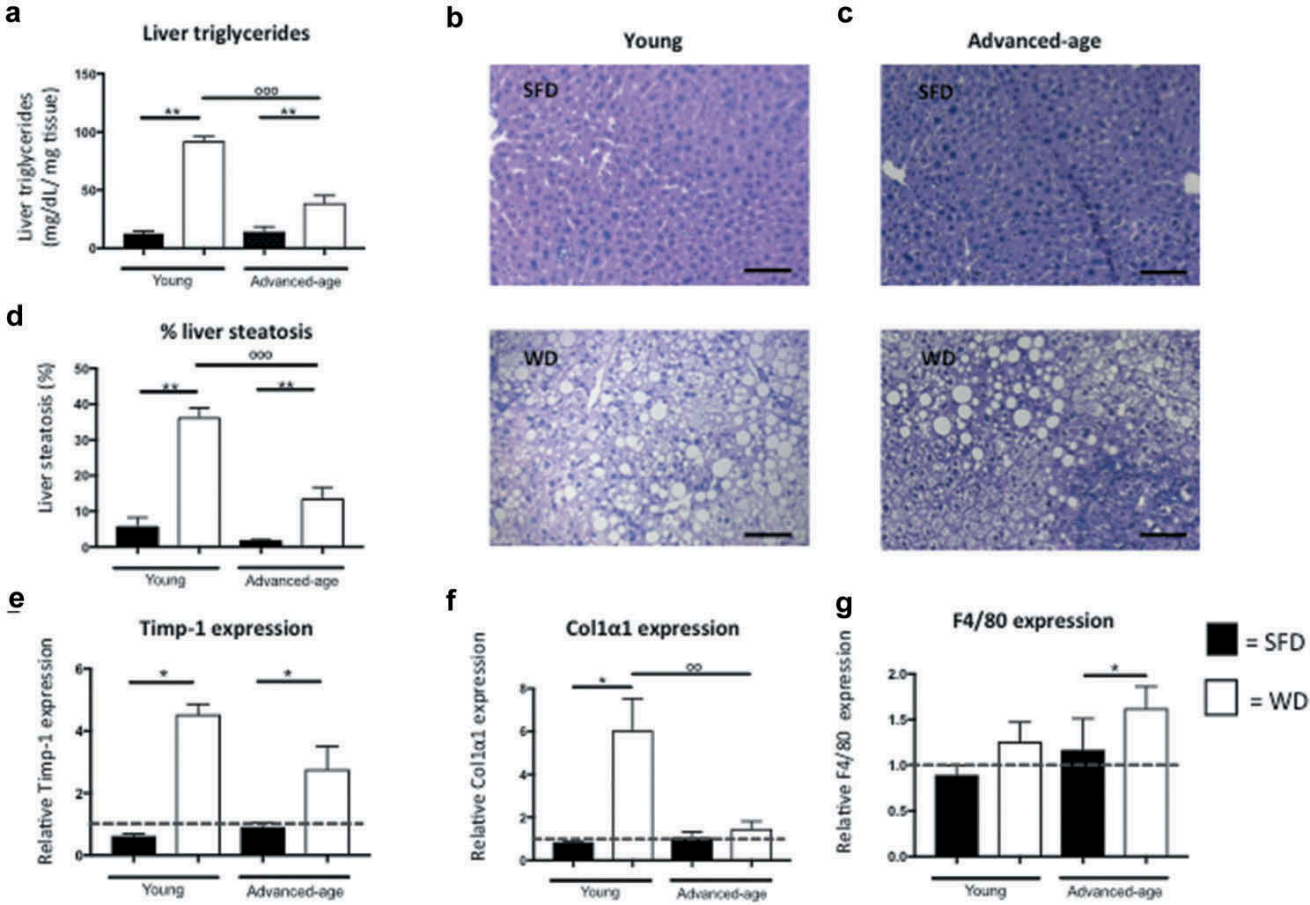
Increased hepatic steatosis but not fibrosis in advanced-age mice. (a) Hepatic triglyceride concentration (mg/dL/mg tissue) in young and advanced-age mice kept on either SFD (black) or WD (white) for 15 weeks. (b-c) H&E stained liver section of a young mouse kept on SFD (top) or WD (bottom) (B) and of an advanced-age mouse kept on SFD (top) or WD (bottom) for 15 weeks (C). Magnification is 200x and scale bar is 100 µm. (d) Quantification of H&E stained liver sections of young and advanced-age mice kept on SFD (black) or WD (white). (e-f) relative hepatic Timp-1 and Col1α1 expression in young and advanced-age mice kept on SFD (black) or WD (white). (g) relative hepatic F4/80 expression in young and advanced-age mice kept on SFD (black) or WD (white). * = p < 0.05 and ** = p < 0.01 in SFD vs WD. ° = p < 0.05, °° = p < 0.01 and °°° = p < 0.001 between young and advanced-age mice kept on the same diet. Fas = TNF receptor superfamily member 6, CD = cluster of differentiation, SFD = standard-fat diet, WD = western-type diet, Timp-1 = tissue inhibitor of matrix metalloproteinase-1, Col1α1 = collagen type 1 α 1.

Unexpectedly, we found that in both age groups plasma triglyceride levels were higher in the SFD groups compared to the WD groups (p = 0.040 for young and p = 0.052 for advanced-age mice). Furthermore, we found that both young groups had higher plasma triglyceride levels as compared to their advanced-age counterparts (p = 0.010 for the SFD and p = 0.002 for the WD) (Table 4). In contrast, hepatic triglycerides were significantly higher in the WD-group as compared to the SFD-group for both age groups (p = 0.008 for young and p = 0.002 for advanced-age mice) (Figure 1(a)). Additionally, we found that hepatic triglycerides were higher in the young WD-group as compared to the advanced-age WD-group. Furthermore, liver weight also was significantly higher for WD-fed as compared to SFD-fed mice in both age groups (p = 0.008 for the young and p = 0.011 for the advanced-age group). In both age groups, H&E-stained liver sections showed significantly more lipid accumulation in the livers of WD groups compared to SFD groups (p = 0.008 for the young and p = 0.005 for the advanced-age mice) (Figure 1(b-f)). Furthermore, lipid accumulation was significantly higher in the young WD-fed mice as compared to their advanced-age counterparts (p = 0.001). The liver enzymes alkaline phosphatase and alanine aminotransferase (ALT) were significantly higher in the WD group compared to the SFD group in the young mice but not in the advanced-age group (Table 4). Furthermore, alkaline phosphatase also was significantly higher in the young WD-fed group as compared to the advanced-age WD-fed group (p = 0.006). No statistical difference was found for aspartate aminotransferase (AST) in either age group (Table 4). Analysis of mRNA levels of several steatosis markers revealed a significantly higher expression of cluster of differentiation 36 (CD36) in livers of young mice fed WD as compared to the SFD controls and in the advanced-age mice a higher expression of tumour necrosis factor (TNF) receptor superfamily member 6 (Fas) in the WD group as compared to SFD controls (p = 0.008 and p = 0.005, respectively) (data not shown). CD36 expression was also higher in livers of young WD-fed mice as compared to advanced-age WD-fed mice (p = 0.001). Furthermore, mRNA levels of the fibrosis markers tissue inhibitor of matrix metalloproteinases-1 (Timp-1) and collagen type 1 α 1 (Col1α1) were significantly higher in the WD group compared to the SFD group in young mice (p = 0.036 for both markers) but not in advanced-age mice (p = 0.068 and p = 0.222, respectively) (Figure 1(g, h)). Col1α1 expression also was significantly higher in the young WD-fed group as compared to their advanced-age counterparts. (0.004). Additionally, in advanced-age but not in young mice, F4/80 expression in liver was significantly higher for WD-fed mice compared to SFD-fed mice (p = p = 0.044 and p = 0.191, respectively) (Figure 1(i)). These results indicate that a WD can induce an increased hepatic lipid accumulation in both age groups, but that in younger mice also higher levels of markers for hepatic fibrosis as well as elevated levels of alkaline phosphatase and ALT are induced. Conversely, advanced-age mice show a higher hepatic expression of F4/80 upon exposure to a western diet.

### Protection against obesity in advanced-age mice might be due to a higher need of energy to combat age-related comorbidities

**Figure 2.**
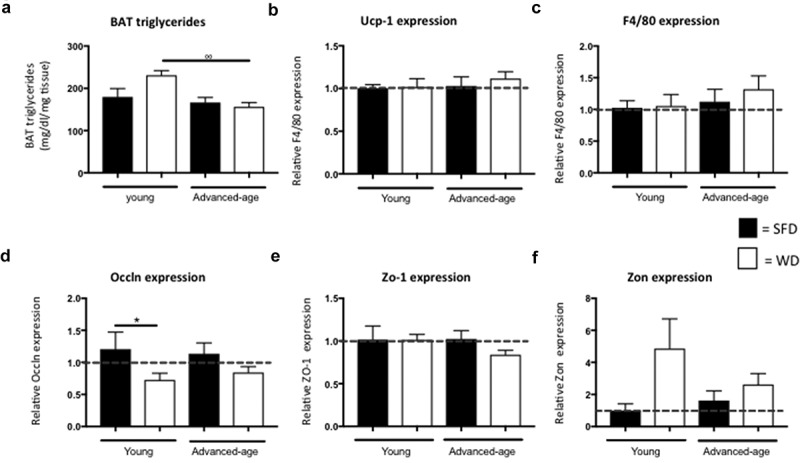
No difference in relative expression of Ucp-1 and F4/80 in BAT and Occln, Zo-1 and Zon in colon of advanced-age mice. (a) Triglyceride concentration in brown adipose tissue. (b-c) Relative expression of Ucp-1 (B) and F4/80 (C) in BAT. (d–f) Relative expression of Occln (D), Zo-1 (E) and Zon (F) in colon. ° = p < 0.05, °° = p < 0.01 and °°° = p < 0.001 between young and advanced-age mice kept on the same diet. SFD = standard-fat diet, WD = western-type diet, Ucp-1 = uncoupling protein- 1, Occln = occludin, Zo-1 = zona occludens-1 and Zon = zonulin.

As advanced-age mice, in contrast to young mice, do not develop obesity upon exposure to a WD, we hypothesized that they might have an excessive functioning of BAT or an impaired fat intestinal absorption. Interestingly, both young and advanced-age mice on a WD showed a higher BAT mass as compared to mice on SFD (p = 0.008 for the young and p = 0.001 for the advanced-age group) (Table 2). BAT mass also was significantly higher in both diet groups of the young mice as compared their advanced-age counterparts (p = 0.037 for SFD and p = 0.001 for WD). However, triglyceride levels were not significantly different between SFD- and WD- fed mice of either age group (Figure 2(a)). BAT-triglycerides were significantly higher in the young WD group as compared to the advanced-age WD- group (p = 0.003). However, no significant difference was found in UCP-1 mRNA levels (Figure 2(b)). Furthermore, a Western blot for UCP-1 also showed no significant difference between groups (data not shown). BAT contains different immune cell populations that can affect BAT-function [12]. Therefore, we analysed F4/80 expression in BAT but found no significant differences between groups (Figure 2(c)).

Analysis of mRNA levels of the intestinal integrity markers occludin (Occln), zona occludens-1 (ZO-1) and zonulin (Zon) in the colon did not show a significant difference in expression between advanced-age SFD- and WD-fed mice (p = 0.075, p = 0.089 and p = 0.387, respectively) (Figure 2(d-f)). In the young mice, however, we did find a higher expression of Zon in the WD-fed mice as compared to the SFD-mice (p = 0.032) suggesting that, in younger mice, intestinal permeability is increased upon obesity-development.

Since almost every animal in the advanced-age groups suffered from age-related comorbidities, we hypothesized that the advanced-age groups might have a higher energy-need to fight of these comorbidities. Therefore, we analysed plasma LDH levels, a surrogate for organ damage. Advanced-age mice in the WD-group indeed had higher LDH-levels as compared to advanced-age mice kept on SFD (p = 0.028). Between the young groups, no statistical differences were found.

Taken together, these results suggest that the protection of advanced-age mice against WD-induced obesity is most likely not due to excessive BAT-functioning or altered intestinal absorption.

## Discussion

In this study, we demonstrated that, in contrast to young mice, advanced-age mice do not develop obesity upon exposure to a WD. Furthermore, we have shown that in advanced-age mice, hepatic lipid accumulation was increased upon WD-exposure without markedly altering liver function. However, higher F4/80 expression does suggest hepatic inflammation in advanced-age mice. Finally, we found that the protection advanced-age mice have against diet-induced obesity was not due to the excessive function of BAT or to an impaired intestinal absorption of fat.

The compositions of the diets used in this study were carefully chosen to avoid ending up with a control diet that consists of >50% carbohydrates or protein. Therefore, the amount of fat and carbohydrates in our control diet still is 30%. This could explain why the mice fed the control diet reached such high body weights. Another explanation why the control mice reach rather high body weights might be that since our control diet consists of 40% protein, the mice develop higher muscle masses.

With age, adiposity gradually increases in humans with a preferential accumulation of visceral fat and a decrease in subcutaneous fat [15]. Studies have shown that homozygous C57BL/6J Clock^Δ19^ mutant mice, a murine model designed to study ageing, develop obesity [16]. Furthermore, the body weight of natural ageing C57BL/6JRj mice increases up to 12 months followed by a decrease up to 24 months [5]. This could explain why in our study, the advanced-age mice do not develop obesity upon western diet exposure.

Pettan-Brewer and Treuting found that in a colony of C57BL/6J mice, 17% of the advanced-age mice displayed seminal vesiculitis [17]. Since we wanted to anticipate on the risk of premature death due to this higher rate of development of age-related comorbidities, we chose n = 10 for the advanced-age mice. For the young mice, we decided on n = 5 since previous studies performed in our lab and existing literature have shown that a WD induces obesity in young adult mice. Furthermore, we expected variability to be much lower in this group because they only rarely develop comorbidities. Indeed, in our study, several mice in the advanced-age group presented with discolouring of the seminal glands, suggesting vesiculitis (data not shown), and higher LDH-plasma levels. LDH is a surrogate marker for organ damage. Therefore, we propose that the advanced-age mice might need the extra energy supplied by the western diet to fight of the development of age-related comorbidities. To adequately assess energy expenditure, a new study using metabolic cages would be necessary.

Since younger mice exposed to a western diet do develop obesity, it would be very interesting to follow up the effects of a long-term exposure to a WD, from 10 weeks up to 24 months.

Ageing in man is associated with hyperglycaemia [18], hyperinsulinaemia [18], hypercholesterolaemia [19] and impaired liver function [20]. However, centenarians have much lower plasma glucose and insulin levels, suggesting that they have a better insulin sensitivity compared to younger individuals [21,22]. In the study by Hemmeryckx et al., 24 month-old mice displayed significantly lower plasma levels of insulin and glucose as compared to their 12 month-old and 10 week-old counterparts, suggesting improved insulin sensitivity [5]. In the present study we found that WD-exposure of 22 month-old mice did not alter insulin and glucose levels in the plasma, resulting in a similar HOMA-index suggesting that upon old age, WD-exposure does not induce insulin resistance.

These results are contradictory to what is known for young animals. In the study of Surwit et al., WD-feeding resulted in an increased body weight, accompanied by fasting hyperglycaemia and hyperinsulinaemia [2]. However, in the present study, we were not able to confirm insulin resistance induced by a WD. This may be due to the genetic and phenotypic differences between C57BL/6 sub-strains concerning responsiveness to a HFD [23].

We noticed that BAT mass was increased upon a 15-week WD loading of advanced-age C57BL/6JRj mice. This was not due merely to an increased lipid accumulation. Peterson *et al*. also reported that BAT weight increased with age in male, but not in female C57BL/6J mice proportional to the body weight change with age [24]. Short-term fat loading of adult 10 weeks old male C57BL/6J mice has previously been shown to enhance the fatty acid combustion potential of BAT, while morbid obesity established after long-term exposure to an HFD renders BAT unresponsive to fat [23]. Ageing per se does not influence the number of F4/80-positive macrophages; however, exposure of adult C57BL/6J mice to a HFD for 8 weeks led to a reduction of macrophages in obese BAT [25]. Furthermore, in humans, ageing is associated with a reduction of BAT mass and activity [26]. However, in our study analysis of mRNA levels of UCP1 and F4/80 revealed no significant differences between advanced-age mice kept on WD or SFD indicating that, in this study, excessive lipid accumulation in BAT or macrophage accumulation does not account for the higher BAT weight or for protection against diet-induced obesity.

In conclusion, we demonstrated that advanced-age mice seem to be protected against diet-induced obesity. However, a western diet did cause an increased hepatic lipid accumulation as well as an increased hepatic F4/80 expression although liver enzymes were not significantly affected. Overall, these findings may be relevant for investigators using mouse models of obesity or ageing.

## Abbreviations

ALT: alanine aminotransferase
AST: aspartate aminotransferase
AT: adipose tissue
BAT: brown adipose tissue
CD36: cluster of differentiation 36
Col1α1: collagen type 1 α 1
Ct: cycle threshold
Fas: tumour necrosis factor receptor superfamily member 6
GN: gonadal
H&E: haematoxylin/eosin
HFD: high-fat diet
HOMA-IR: homeostatic model assessment of insulin resistance
Ip: intraperitoneal
LDH: lactate dehydrogenase
Mes: mesenteric
Occln: occludin
SC: subcutaneous
SFD: standard-fat diet
Timp-1: tissue inhibor of matrix metalloproteinases-1
TNF: tumour necrosis factor
Ucp-1: uncoupling protein 1
WD: western-type diet
Zo-1: zona occludens-1
Zon: zonulin
